# A Rare Anastomosis Between the Internal and the External Jugular Veins: A Case Report

**DOI:** 10.7759/cureus.55212

**Published:** 2024-02-29

**Authors:** Anastasia I Bekyarova, Adelina V Velikova, Nikol V Atanasova, Marin D Zhelezov

**Affiliations:** 1 Department of Anatomy and Cell Biology, Medical University of Varna, Varna, BGR

**Keywords:** neck dissection, anatomical variation, external jugular vein, internal jugular vein, anastomosis

## Abstract

Usually, the external jugular vein (EJV) is located superficially over the sternocleidomastoid muscle and joins the subclavian vein or the venous angle. The internal jugular vein (IJV) lies deeply in close relation with the common carotid artery and vagus nerve, enveloped by the carotid sheath. Normally, there is no direct connection between those vessels. During a routine neck dissection, we found a rare anastomosis between IJV and EJV. The anastomosis was localized on the level of the cricoid cartilage. It was approximately 1 cm long, with the diameter of the lumen being 0.3 cm. There was no obstruction along the length of the vessel. The direction was oblique and followed the blood flow from IJV to EJV. The observed variation has high clinical importance related to numerous procedures executed in the neck region, such as placement of hemodialysis catheter in patients with renal failure, insertion of central venous line in the care of critically ill patients, and radical neck dissections.

## Introduction

The superficial veins of the neck are represented mainly by the external and anterior jugular veins. They take part in the venous drainage from the soft tissues around the auricula and the mental region. The external jugular vein (EJV) is formed by the convergence of the posterior auricular vein and the posterior branch of the retromandibular vein. The former communicates with the mastoid emissary vein, thus playing a role in cerebral venous drainage, and the latter drains the venous blood from the face and the scalp. Usually, EJV travels superficially down the neck over the sternocleidomastoid muscle (SCM) and joins the subclavian vein between the medial and middle third of the clavicle or ends in the venous angle. The largest deep vein of the neck is the internal jugular vein (IJV), which lays inside the carotid sheath together with the common carotid artery and vagus nerve. It is a direct continuation of the sigmoid sinus, thus draining venous blood from the cranial cavity, but also receiving blood from some extracranial veins of the head. Normally, the external and IJVs do not anastomose with each other, only in very rare cases [1].

## Case presentation

**Figure 1:**
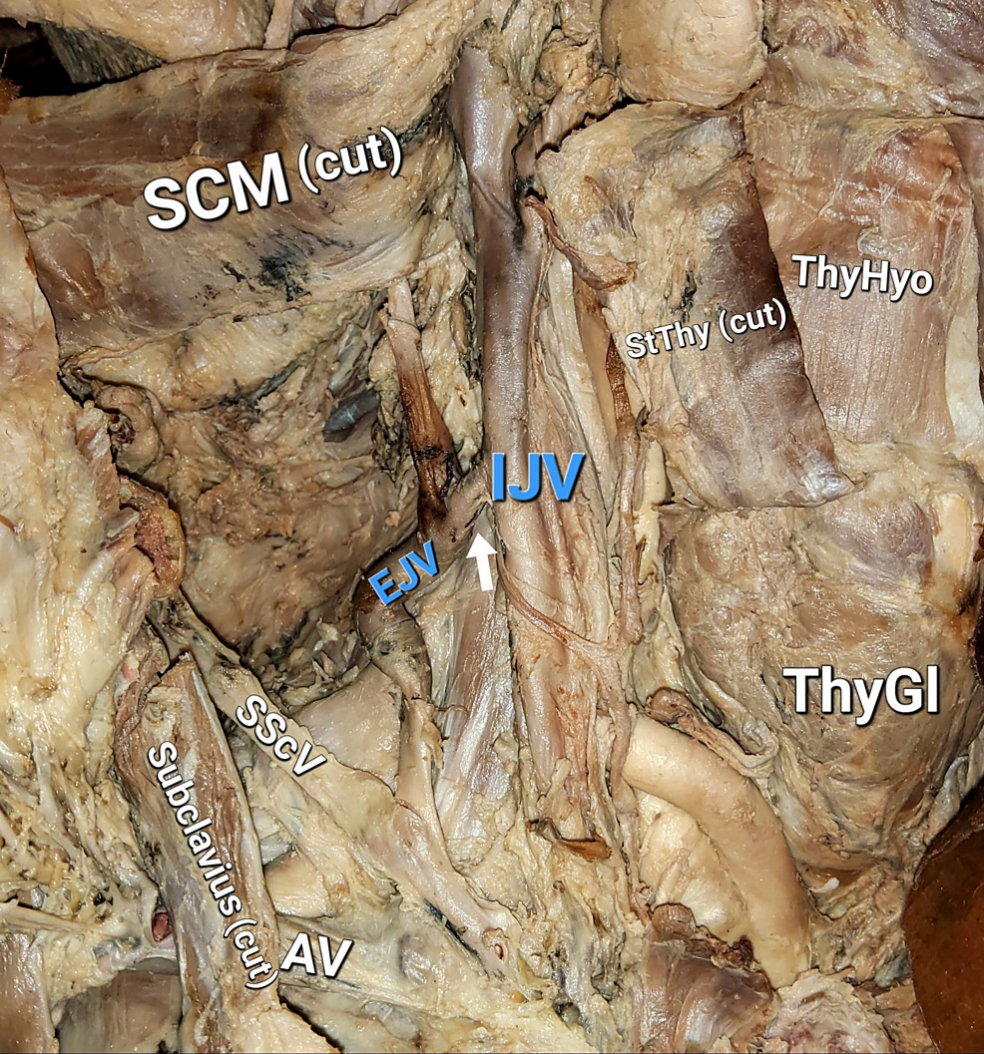
Photograph of the specimen Arrow: anastomosis, IJV: internal jugular vein, EJV: external jugular vein, SScV: suprascapular vein, AV: axillary vein, SCM: sternocleidomastoid muscle, StThy: sternothyroid muscle, ThyHyo: thyrohyoid muscle, ThyGL: thyroid gland

During a routine neck dissection of an adult female cadaver in the dissection halls of the Department of Anatomy and Cell Biology at the Medical University of Varna, Varna, Bulgaria, we found persisting anastomosis between the right external and IJVs. The vessels, their tributaries, and surrounding structures were carefully followed and dissected. Measurements were taken, and the findings were photographed (Figure 1).

**Figure 2:**
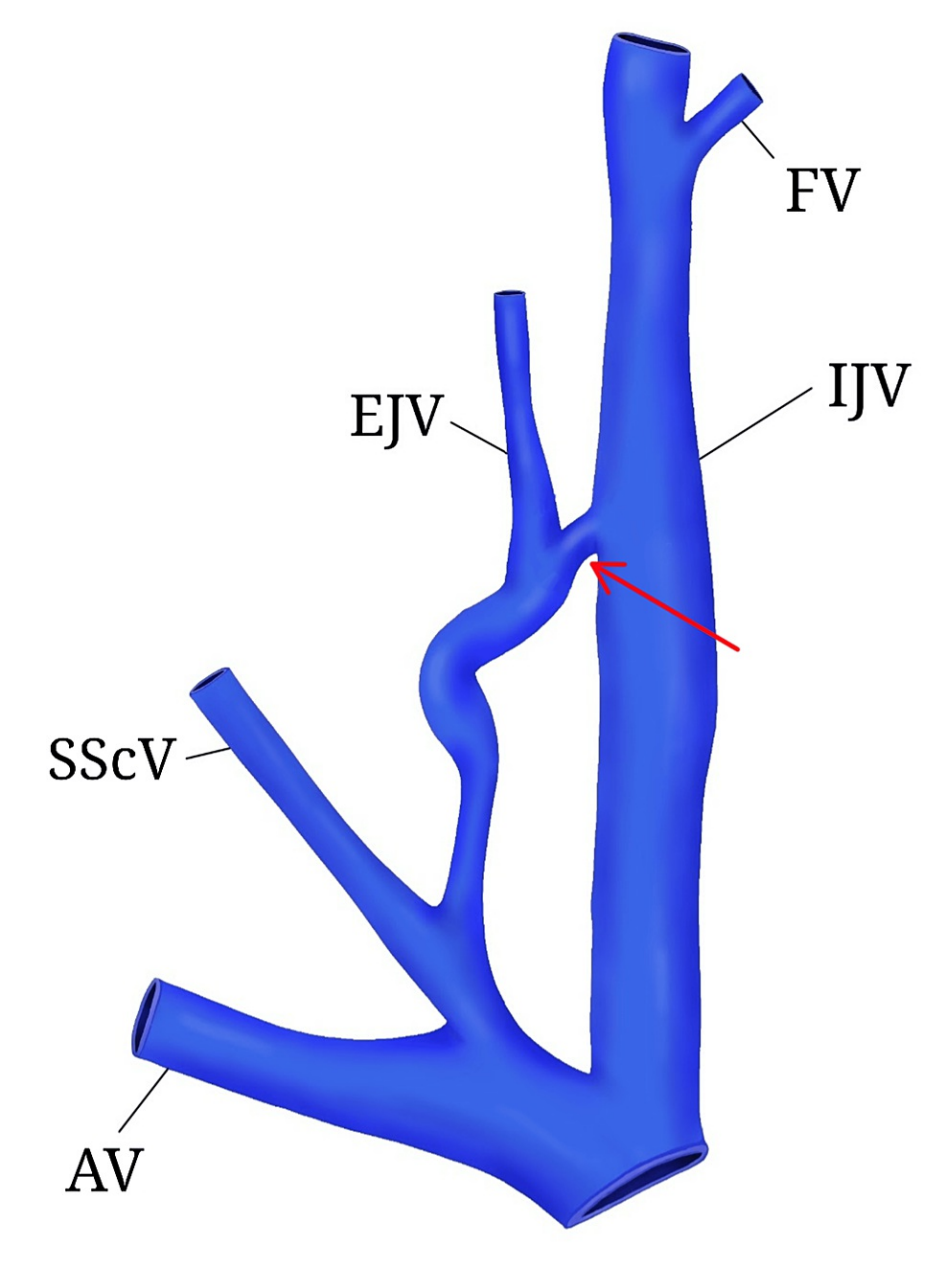
Digital model of the subjects of interest created with Sketchbook (illustration software) Anastomosis between EJV and IJV (red arrow), IJV: internal jugular vein, EJV: external jugular vein, SScV: suprascapular vein, AV: axillary vein, FV: facial vein. Image credit: Anastasia I. Bekyarova

The formation of the vessels showed no departure from the norm. The anastomosis was localized on the level of the cricoid cartilage, behind the posterior margin of the SCM, and was 1 cm long, with a diameter of 0.3 cm. Its direction was oblique and followed the blood flow from IJV to EJV. Caudally to the anastomosis, a dilated curve was observed in the EJV with a length of 4.5 cm and diameter of 0.7 cm. This region was occluded, probably during the preservation process. Before its termination in the well-developed right suprascapular vein (instead of the right subclavian), the EJV seemed to recover its morphology, course, and size (Figure 2).

The anastomosis was fully functional. No obstacles were found to restrict the blood flow through the vessel.

## Discussion

The EJV is known to exhibit numerous variations regarding its presence, formation, course, tributaries, communications, and termination [2,3].

In a study that aimed to describe the variant anatomy of EJV, the neck regions of 53 Kenyan cadavers were dissected, and the results were as follows: normal anatomy was noted in 82% of the cases, and in 15% of the male cadavers, the vein was absent unilaterally, mainly from the right side. None of the female cadavers presented with such a variation. The EJV was duplicated in two cases and terminated into the IJV in 7.7% of the cases [4].

Communications between the internal and the EJVs were described in several case reports [5-10]. In some of them, the anastomosis represents a longer (≥2.5 cm) vessel and usually arises from EJV, runs downwards, and reaches IJV. In our case, the direction is from IJV to EJV, and the length is about 1 cm. Patera et al. report similar but horizontally situated anastomosis [8].

Such differences in the anatomy of the venous system are related to the embryological development of the structures and the formation of new anatomical variants. In the early stages of human development, the anterior cardinal vein is responsible for the venous drainage of the cephalic region [11]. Later on, its cranial portion regresses in size and turns into IJV. According to recent data, capillary plexuses in the face give rise to the EJV and become connected to the newly formed vessel. Soon, the venous network of the upper limb joins it too, thus determining the formation of the subclavian vein, the venous angle, and the brachiocephalic vein [12,13]. We suggest that under the stimulation of specific factors through the process of angiogenesis-expansion and remodeling of the vascular system by sprouting-an anastomosis has occurred between IJV and EJV.

The clinical importance of knowing about this possible communication is seen in various situations and clinical cases. As the IJV drains deoxygenated blood from the cranial cavity, such anastomosis may increase the risk of infections spreading from the face and neck to the interior of the cranium [5]. Moreover, the fascial spaces in the head and neck are potential sites where infections may spread [14]. Note that the anastomosis connects the carotid sheath and superficial cervical fascia, which envelops the platysma muscle. Awareness during radical neck surgeries might prevent unnecessary bleeding and injuries [8,15]. Attention must be paid during the placement of a central venous catheter (CVC) in the IJV to avoid complications [5], such as vascular injury, infection, and misplacement [16]. Additionally, hemodialysis catheter placement in the subclavian vein shows more favorable results and fewer complications in comparison to the IJV [17]. The presence of such anastomosis would further increase the existing risk of early and late hemodialysis catheter-related complications in the case of IJV catheterization.

## Conclusions

In conclusion, we suggest that knowing about possible communications between the major vessels in the neck region might be a privilege for clinicians working in different medical fields who perform various procedures in this anatomic area. That may help avoid further complications and lead to a better outcome for the patient.
